# Transcriptomic comparison on the mechanism of action of four major constituent cannabinoids in hemp extract

**DOI:** 10.1186/s42238-026-00432-w

**Published:** 2026-04-29

**Authors:** Xiugong Gao, Miranda R. Yourick, Kayla Campasino, Yang Zhao, Estatira Sepehr, Cory Vaught, Robert L. Sprando, Jeffrey J. Yourick

**Affiliations:** Division of Toxicology, Office of Chemistry and Toxicology, Office of Laboratory Operations and Applied Science, Human Foods Program, U.S. Food and Drug Administration, 8301 Muirkirk Rd, Laurel, MD 20708 USA

**Keywords:** Cannabinoid, Cannabidiol (CBD), Cannabichromene (CBC), Cannabigerol (CBG), Cannabinol (CBN), Mechanism of action, Toxicity

## Abstract

**Background:**

A variety of health benefits have been claimed, but not scientifically confirmed, for cannabidiol (CBD) and other nonintoxicating cannabinoids in hemp extracts such as cannabichromene (CBC), cannabigerol (CBG), and cannabinol (CBN). On the other hand, CBD has been reported to cause hepatotoxicity in clinical trials and research studies, while little is known about the safety of other cannabinoids. In the current study, we set out to understand the mechanism(s) of action of these cannabinoids, beneficial or adverse, through a comprehensive functional analysis of a recently published transcriptomic dataset.

**Methods:**

iPSC-derived hepatocytes were exposed to 40 µM CBC, 20 µM CBD, 40 µM CBG, and 10 µM CBN, respectively, for 24 h. Gene expression changes were profiled using Affymetrix GeneChip PrimeView Human Gene Expression Arrays. Functional analysis was conducted using Ingenuity Pathway Analysis.

**Results:**

Each compound impacted a unique list of canonical pathways, upstream regulators, diseases and biological functions, toxicity functions, and gene interaction networks with distinctive activation/inhibition patterns. Overall, the cannabinoids were predicted to affect metabolism and to have some beneficial effects on cardiovascular disease but adverse effects on the neural system. In addition, CBC and CBN, similar to but more potently than CBD, displayed liver toxicity and the potential to cause cancer while CBG protected from these adverse effects.

**Conclusion:**

This study provides a comprehensive comparison across the four cannabinoids and points to the directions for further research on the therapeutic effects and potential toxicities associated with long-term use of these cannabinoids.

**Supplementary Information:**

The online version contains supplementary material available at 10.1186/s42238-026-00432-w.

## Introduction

The 2018 U.S. Federal Agriculture Improvement Act (Farm Bill) exempted hemp from the controlled substances list. Since then, consumer products derived from hemp extract have been aggressively marketed in the US and other parts of the world through various channels (Mead 2019). Hemp is a class of botanical cultivars of the *Cannabis sativa* L. plant with lower concentrations (≤ 0.3% by dry weight) of Δ^9^-tetrahydrocannabinol (THC), the primary psychoactive component, grown specifically for industrial or medicinal use (Erickson 2019; Mead 2019). More than 500 compounds have been found in *C. sativa* including cannabinoids, non-cannabinoid phenols, flavonoids, terpenes, alkaloids, and others (ElSohly et al. 2017; Radwan et al. 2021).

Cannabinoids are C_21_ terpeno-phenolic compounds specific to *Cannabis*. At least 125 varieties of cannabinoids have been isolated from the plant (Filipiuc et al. 2021; Radwan et al. 2021). Among them, cannabidiol (CBD) is the most abundant in hemp. Despite being structurally similar to THC, CBD lacks the psychoactive effects; however, it is widely touted as an effective therapeutic for a diverse array of health conditions, including epilepsy, anxiety, pain, inflammation, schizophrenia, various substance use disorders, post-traumatic stress disorder, and cancers (Mashabela and Kappo 2024; Seltzer et al. 2020; Sholler et al. 2020). The U.S. Food and Drug Administration (FDA) has currently only approved CBD for the treatment of certain rare forms of epilepsy (Ryan 2020). Many other purported health benefits of CBD, such as anti-inflammatory and anxiolytic effects, have not yet been well established (Bridgeman and Abazia 2017). On the other hand, CBD has been associated with elevated liver enzymes and potential hepatotoxicity in clinical trials for Epidiolex approval (Lo et al. 2023). Over the past years, a growing body of publications appeared in the literature investigating liver toxicity (and other organ toxicities) of CBD and CBD-containing hemp extract in animal models (Clewell et al. 2023; Costa et al. 2025b; Dehner et al. 2025; Dziwenka et al. 2020, 2023a, 2023b; Ewing et al. 2024; Henderson et al. 2023; Kutanzi et al. 2020; Pinto et al. 2024; Pintori et al. 2024; Polanska et al. 2023) or using in vitro hepatoxicity models (Campasino et al. 2024; Chen et al. 2024; Gao et al. 2024; Li et al. 2023; Striz et al. 2024). However, the mechanism of CBD-induced hepatotoxicity has not been well understood so far.

Several other cannabinoids were also found in substantial amounts in hemp extracts, including cannabichromene (CBC), cannabigerol (CBG), and cannabinol (CBN) (ElSohly et al. 2017; Filipiuc et al. 2021; Pertwee 2006). Both CBC and CBG are non-psychoactive while CBN is mildly psychoactive (Martínez et al. 2020). Similar to CBD, a wide array of health benefits have been suggested for these cannabinoids, such as antimicrobial, anti-inflammatory, anticonvulsant, antinociceptive, neuroprotective, and anti-tumor, yet more studies are needed to confirm these effects (Blebea et al. 2024; Li et al. 2024; Sampson 2021; Sepulveda et al. 2024). However, only a dearth of studies has addressed the hepatotoxicity of these cannabinoids (Bailey et al. 2022; Dalterio et al. 1986; Polanska et al. 2023).

A large number of non-FDA approved cannabis-derived products on the market are formulated with “full spectrum” hemp extract enriched in CBD but also contains a variety of other cannabinoids and some non-cannabinoid compounds as well (Miller et al. 2022; Perez-Vilar et al. 2023). In a previous study, we conducted a transcriptomic concentration response study in iPSC-derived hepatocytes on an ethanol preparation of hemp extract and its four major constituent cannabinoids, CBD, CBC, CBG, and CBN (Gao et al. 2024). Apart from cannabidivarin (CBDV), these were the most abundant cannabinoids in the hemp extract that was used in the study, which had the following composition (% w/w): 94.25 CBD, 2.80 CBC, 1.84 CBG, 0.72 CBDV, and 0.37 CBN (Gao et al. 2024). We compared their potency based on their respective transcriptomic point of departure (tPOD) concentrations and explored their mechanisms of action through identifying the common pathways and biological processes impacted by their exposures. In the current study, we further analyzed the transcriptomic data and delved deeper into the canonical pathways, upstream regulators, diseases and biological functions, toxicity functions, and their interaction networks impacted by each of the four cannabinoids. In addition to the pathways, functions, and networks commonly impacted by all the cannabinoids, we also identified distinct features for each compound. Despite some therapeutic potential, a variety of adverse effects such as liver toxicity and cancer development and progression were also predicted for these cannabinoids.

## Materials and methods

### Cell culture and cannabinoids exposure

Commercial iPSC-derived hepatocytes (iCell Hepatocytes 2.0) were obtained from FUJIFILM Cellular Dynamics (Madison, WI). Cells were cultured following the manufacturer’s protocol in 24-well cell culture plates coated with rat tail collagen type I at a cell density of 3 × 10^5^ cells/cm^2^. Cells were ready for use between days 5–8 after seeding.

Reference standard CBD (Item No. 90080), CBG (Item No. 15293), and CBN (Item No. 25495) were purchased from Cayman Chemical (Ann Arbor, MI). Reference standard CBC (NDC No. 51634–2180–09) were purchased from Purisys (Athens, GA). A stock solution of 40 mM for each cannabinoid (CBD, CBC, CBG, or CBN) was prepared in dimethyl sulfoxide (DMSO) as described previously (Zhao et al. 2023) and stored at − 80 °C until use. Cannabinoid exposure was carried out on day 6 of cell culture. Cells were treated with purified cannabinoids in triplicates for each treatment group. Immediately before exposure, the stock solution was further diluted in DMSO to 200 × of the nominal cannabinoid concentrations. Treatment solutions were prepared by diluting the 200 × solutions in maintenance medium to the final concentrations, maintaining a DMSO concentration of 0.5% across all treatments. Cells of the vehicle control groups were treated with maintenance medium containing 0.5% (v/v) DMSO only. The exposure was conducted at 37 °C, 5% CO_2_ for 24 h.

### RNA extraction for transcriptomic profiling

Cells were lysed in RLT buffer (Qiagen, Valencia, CA) supplemented with 1% (v/v) β-mercaptoethanol at the end of the exposure and the lysates were stored at − 80 °C until RNA extraction. The lysates were thawed on ice and homogenized using QIAshredder (Qiagen). Total RNA was purified from the cell lysates using EZ1 Advanced XL automated RNA purification instrument (Qiagen) with the EZ1 RNA Cell Mini Kit (Qiagen), and instructions from the manufacturer were followed. An on-column DNase digestion step was included to remove contaminating genomic DNA. Total RNA concentration and purity (260/280) were subsequently measured using a NanoDrop 2000 UV–vis spectrophotometer (NanoDrop Products, Wilmington, DE). RNA quality was further checked using the Agilent 2100 Bioanalyzer (Agilent, Santa Clara, CA) with the RNA 6000 Nano Reagent Kit (Agilent) to obtain the RNA integrity number (RIN).

### RNA processing and microarray experiment

All reagents and instruments used in the microarray experiment were obtained from Affymetrix (Santa Clara, CA). Total RNA samples were processed using the GeneChip 3' IVT PLUS Reagent Kit and hybridized onto GeneChip PrimeView Human Gene Expression Arrays following protocols from the manufacturer. Briefly, single-stranded complementary DNA (cDNA) was generated from 100 ng total RNA using reverse transcriptase and a T7-linked oligo(dT) primer, which was then converted to double-stranded cDNA using DNA polymerase and RNase H. Subsequently, complementary RNA (cRNA) was synthesized through in vitro transcription (IVT) with biotinylated UTP and CTP, using T7 RNA polymerase as the enzyme and the second strand of the double-stranded cDNA as the template.

The biotin-labeled cRNA was then purified and a fraction of 12 µg was fragmented by Mg^2+^ at 94 °C. Fragmented cRNA was then hybridized onto the microarray chips in the GeneChip Hybridization Oven 645 at 45 °C for 16 h. After hybridization, the microarray chips were stained and washed on the GeneChip Fluidics Station 450. Finally, the chips were scanned using the GeneChip Scanner 3000 7G, and the scanned image (DAT) files were further preprocessed using Affymetrix GeneChip Command Console software (v. 4.0) to produce cell intensity (CEL) files.

### Microarray data processing and analysis

The CEL files were imported into the Affymetrix Transcriptome Analysis Console (TAC) software (v.4.0). Arrays were quality-checked prior to further data processing and analysis, and potential outliers were removed. The robust multi-chip average (RMA) algorithm integrated in the software was used to summarize values of individual probes belonging to one probeset in the CEL files, which consists of three steps: 1) background adjustment; 2) quantile normalization; and 3) summarization. Unsupervised principal component analysis (PCA) on normalized data from all samples and one-way analysis of variance (ANOVA) to identify differentially expressed genes (DEGs) were then performed. For each comparison between two experimental groups, the selection of DEGs was based on the fold change (FC) of each annotated gene coupled with its corresponding false discovery rate (FDR) value.

### Functional analysis

Functional analysis was conducted using the Ingenuity Pathway Analysis (IPA) software (v145030503) from Qiagen. A list of DEGs (FDR adjusted *p*-value < 0.05 and |FC|≥ 1.5) for each cannabinoid at the selected concentration (as described in the main text) was uploaded into the program, and canonical pathways, upstream regulators, diseases and bio functions, tox functions, and biological themes networks (graphical summary) were subsequently analyzed using default settings. The analyses were conducted either individually for each compound using Core Analysis or simultaneously for all compounds using Comparison Analysis.

## Results

### Concentration selection for the cannabinoids

In a previous study (Gao et al. 2024), we conducted a concentration response transcriptomic profiling of the four cannabinoids using microarrays in the concentration range of 0.1 µM to 20 µM (for CBD and CBN) or 40 µM (for CBC and CBG). The higher concentration (40 µM) was not used for CBD or CBN to avoid overt cytotoxicity. Three replicates were obtained for each compound at each concentration, except for CBG at 20 µM where only two replicates were obtained.

For an objective and meaningful comparison between the different cannabinoids in the context of functional analysis, instead of using an identical concentration for all the compounds, we selected a concentration that was the closest to the IC_10_ value for each compound; in other words, we compared the functional impact of the compounds at approximately the same cytotoxicity level. Therefore, based on the IC_10_ values reported previously (Gao et al. 2024), the following concentrations were chosen for functional analysis and comparison: CBC, 40 µM; CBD, 20 µM; CBG, 40 µM; and CBN, 10 µM.

Using in silico prediction, Liu and Sprando (2023) reported that 30-mg oral CBD exposure in a 75-kg individual yields a liver C_max_ of 86.5 nM, which is about 14.4-fold of the predicted plasma C_max_ (6.0 nM). In addition, the highest observed clinical plasma concentrations were found to be between 1 and 3 μM (Chan and Duncan 2021; Contin et al. 2021; Ohlsson et al. 1986). Assuming that the relationship between plasma C_max_ and liver C_max_ is linear, the highest observed clinical plasma CBD concentrations of 1–3 μM predicts a liver C_max_ of approx. 14–43 μM. Data on clinical plasma concentrations for CBC, CBG, and CBN are currently unavailable; however, based on the predictions by Liu and Sprando (2023), at the same exposure level (0.4 mg/kg-bw), liver C_max_ values for both CBC and CBN, 95.2 nM and129.2 nM, respectively, are higher than that of CBD (86.5 nM), and liver C_max_ for CBG (70.0 nM) is only slightly lower than that of CBD. Therefore, based on these values, the concentrations selected for the four cannabinoids in the current study, as stated above, are considered physiologically attainable.

### Overview of the transcriptomic data and functional analyses

**Fig. 1:**
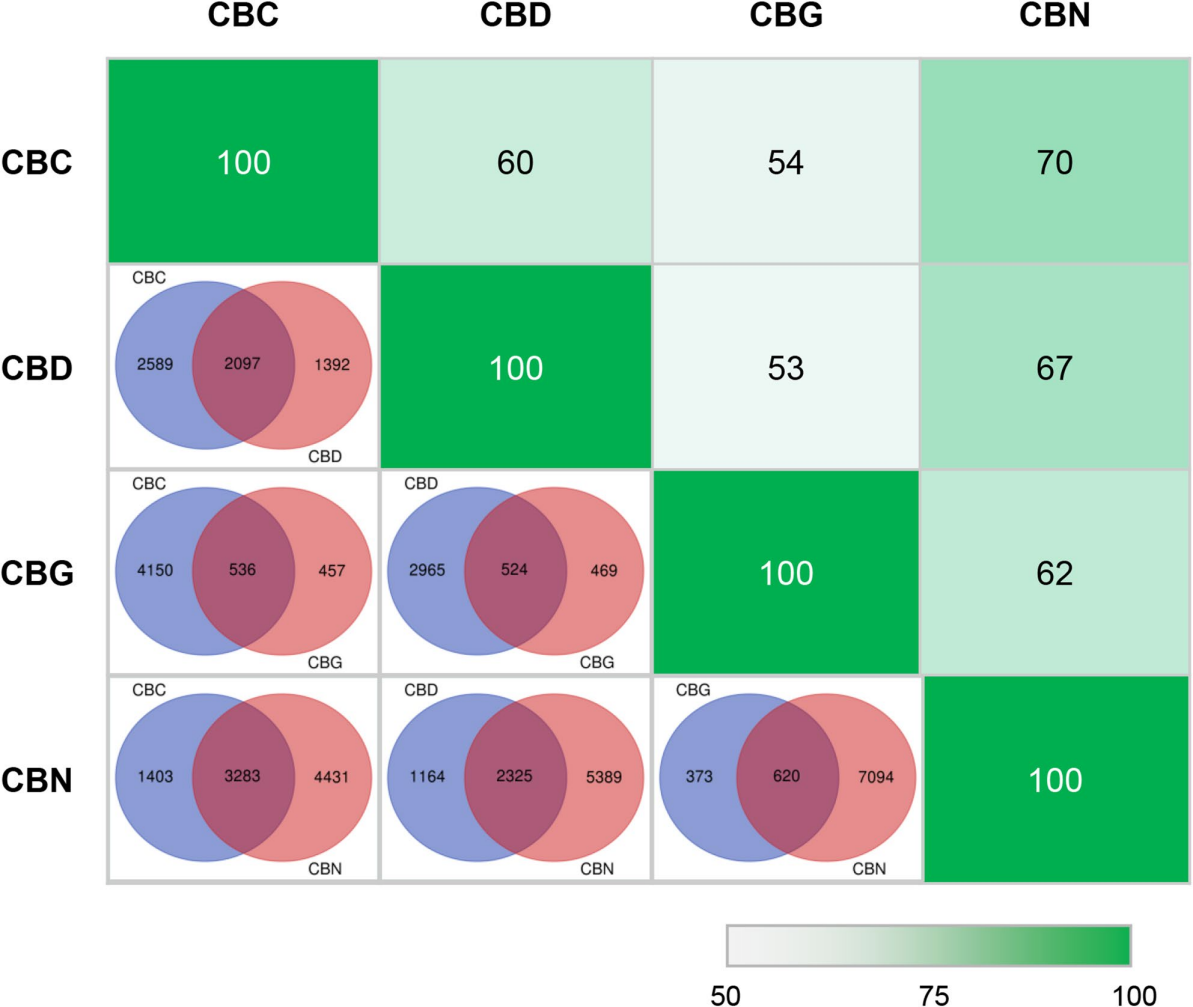
Similarities between the DEGs induced by the different cannabinoids. Overlapping of the DEGs between each pair of compounds is illustrated by the Venn diagrams at the bottom left corner. The overlap coefficients are shown at the top right corner. Overlap coefficient between *A* and *B* was calculated as: overlap (*A*, *B*) =|*A* ∩ *B*|/min(|*A*|, |*B*|)

A total of 4686, 3489, 993, and 2821 DEGs were identified for CBC, CBD, CBG, and CBN, respectively, at the selected concentrations described above. Similarities between the DEGs induced by the different compounds are shown in Fig. 1. For each possible comparison between two compounds, an overlap coefficient was calculated. There were generally moderate agreements among the four cannabinoids, with overlap coefficients between any pair ranging 53–70%. CBC and CBN showed the highest similarity (70%), while CBD and CBG bore the least similarity (53%).

**Fig. 2:**
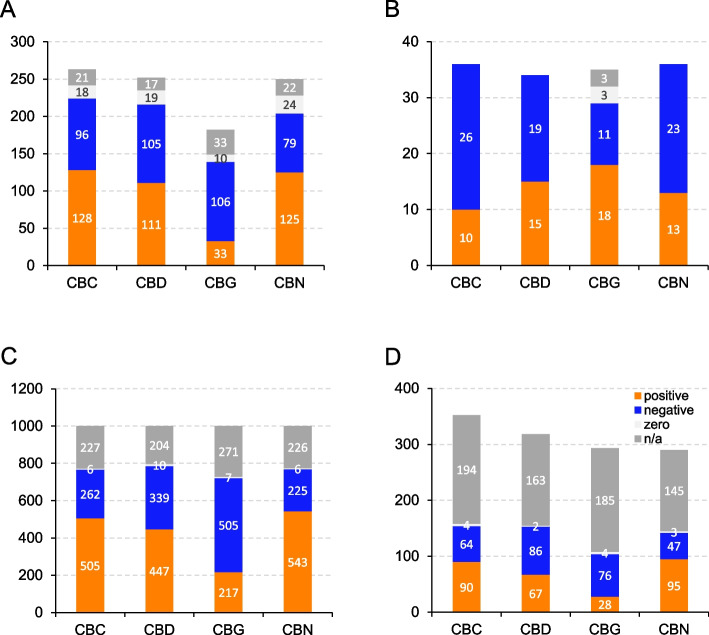
Overview of the IPA output on canonical signaling pathways (**A**), nuclear receptors (**B**), diseases and bio functions (**C**), and tox functions (**D**). The activation/inhibition statuses (*z*-score) are color-coded as shown in the legend and the numbers of terms in each category are displayed in the figure

Functional analysis was conducted using IPA to identify canonical pathways, upstream regulators, diseases and biological functions, toxicity functions, and networks impacted by exposure to each of the four cannabinoids. Figure 2 shows the overall results for each of the analysis types, which will be described in more detail in the sections that follow.

### Canonical signaling pathways

Canonical pathways overrepresented by the DEGs of each compound were first analyzed. The canonical pathways in IPA include three categories: metabolic pathways, Reactome pathways, and signaling pathways. We focused our analysis and comparison on signaling pathways only, as these might be more indicative of the mode of action of the chemicals. Significant (*i.e.*, overrepresented) pathways were identified for each of the cannabinoids using a loose criterion of Fisher’s exact test* p*-value < 0.5. A total of 263, 252, 182, and 250 pathways were identified for CBC, CBD, CBG, and CBN, respectively. IPA also predicts the activation/inhibition states of the identified pathways using a *z*-score calculated based on the directionality of the expression changes of the DEGs. The complete lists of enriched pathways with their activation/inhibition states for each cannabinoid are included in Supplementary Table 1 and summarized in Fig. 2A. CBC, CBD, and CBN had similar numbers of activated (*z* > 0) and inhibited (*z* < 0) pathways; in comparison, CBG had much less activated pathways than the other three, although the inhibited pathways were approximately the same.

**Fig. 3:**
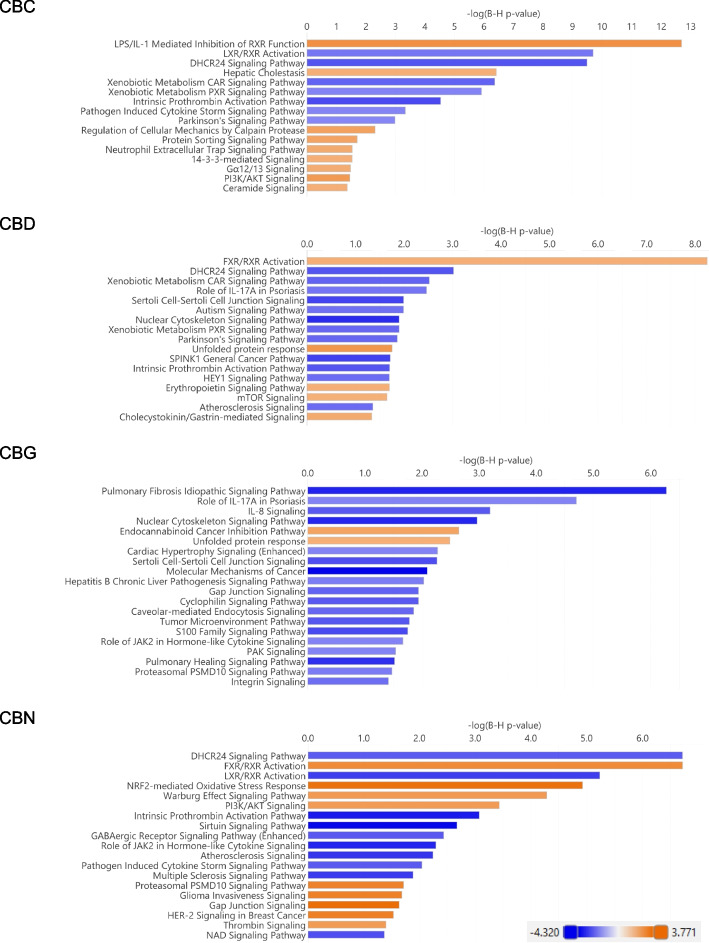
Canonical signaling pathways identified for CBC, CBD, CBG, and CBN using B-H adjusted *p*-value < 0.05 and |*z*|> 2.0. Each pathway is represented by a horizontal bar with the name shown on the left and length equivalent to -log(B-H *p*-value). The activation/inhibition state (*z*-score) of the pathway is indicated by the color of the bar with orange representing activation and blue inhibition. The color scale is shown at the bottom right corner

**Fig. 4:**
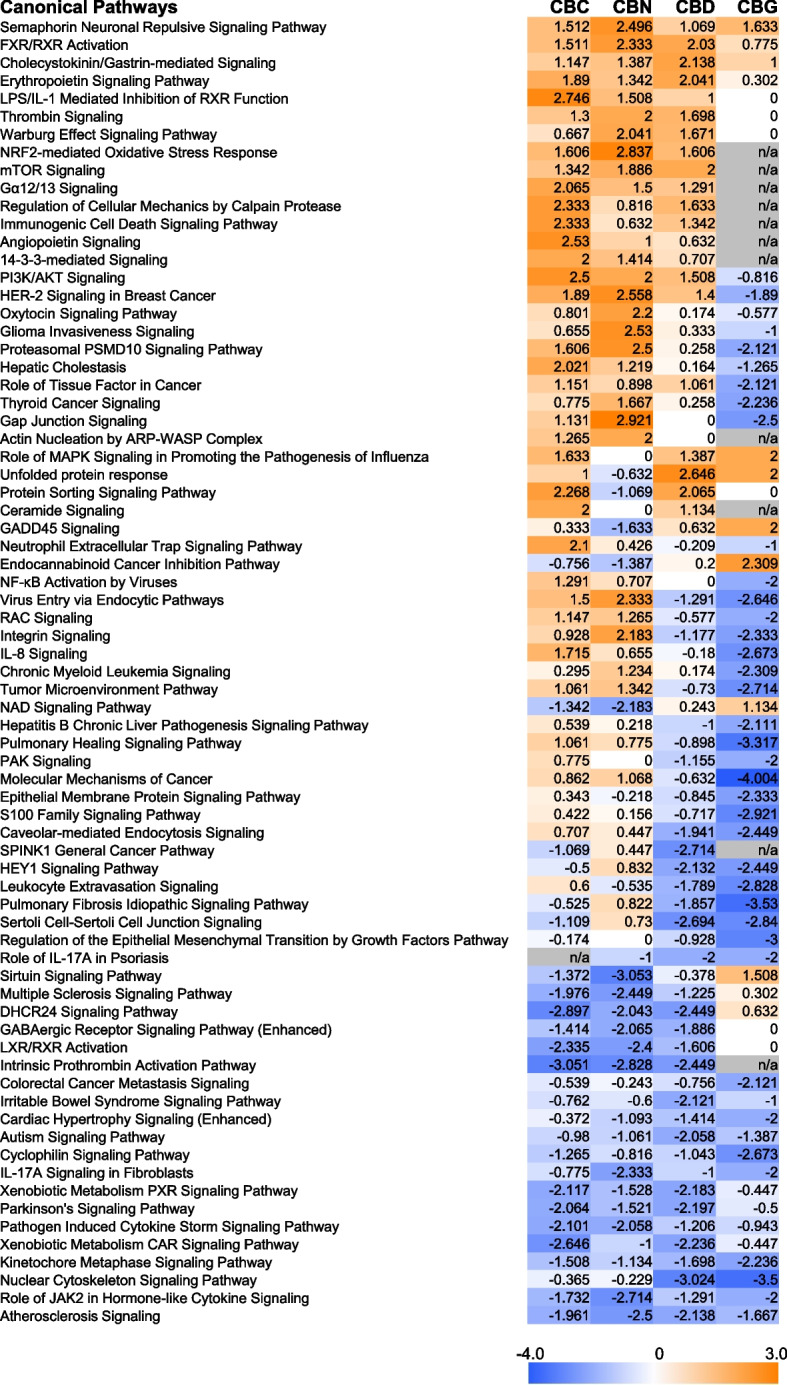
Top 72 signaling pathways that were impacted by all the four cannabinoids (B-H adjusted *p*-value < 0.05), with at least one compound having |*z*|> 2.0. For each pathway, the activation/inhibition state (*z*-score) following exposure to a compound is indicated and color-coded using a scheme as shown at the bottom right corner

For a more simplified comparison, we applied more stringent criteria of Benjamin-Hochberg (B-H) adjusted *p*-value < 0.05 and absolute *z*-score > 2.0. Totally 16 (9 activated/7 inhibited), 17 (5/12), 20 (2/18) and 19 (9/10) pathways were identified for CBC, CBD, CBG, and CBN respectively (Fig. 3). A compilation of all these pathways with *z*-scores shown for all the four cannabinoids (at least one compound having |*z*|> 2.0) is found in Fig. 4. Overall, there were remarkable similarities in the activation/inhibition patterns of the enriched pathways among CBC, CBD and CBN. In contrast, CBG bore little similarity with the other three cannabinoids. Four pathways were activated and 14 were inhibited by all the cannabinoids. Ten pathways were activated and three were inhibited by CBC, CBD and CBN only, with the state in CBG being neither activated nor inhibited (*z* = 0), or unpredictable (n/a). Further, eight pathways were activated by CBC, CBD and CBN but inhibited by CBG, and three vice versa. It was also noted that while the numbers of activated *vs*. inhibited pathways were roughly comparable for CBC, CBD or CBN, the majority of the pathways with known activation/inhibition status (44 out of 56) were inhibited by CBG.

### Upstream nuclear receptors

An upstream regulators analysis was then conducted for the cannabinoids. IPA includes a wide array of upstream regulators broadly categorized into genes, RNAs, proteins, drugs and chemicals. We focused the analysis on (ligand-dependent) nuclear receptors only. Nuclear receptors are a type of transcription factor that are activated by the binding of specific molecules (*i.e.*, ligands), and play a crucial role in regulating a wide range of physiological processes, including reproduction, development, and metabolism (Frigo et al. 2021). Identifying nuclear receptors impacted by cannabinoid exposure may provide critical insights into the mode of action of the cannabinoids. Out of the 48 nuclear receptors found in humans, approximately equal numbers were significantly (*p* < 0.05) impacted by the four cannabinoids – 36, 34, 35 and 36 by CBC, CBD, CBG and CBN, respectively. However, the identities (Supplementary Table 2) and activation states (Fig. 2B) of the nuclear receptors varied among the compounds, with CBC, CBD and CBN bearing some similarities while CBG being quite different to the other three compounds.

**Fig. 5:**
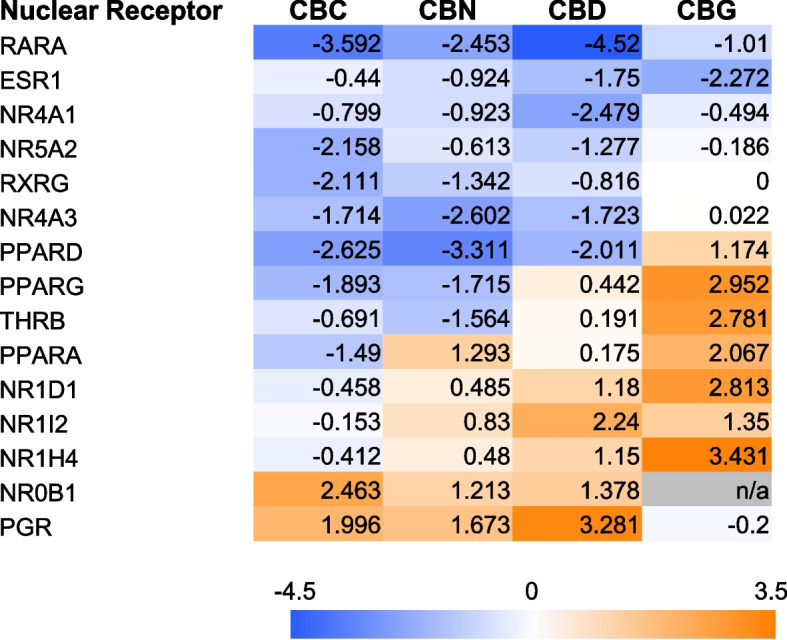
Top 15 nuclear receptors identified for CBC, CBD, CBG, and CBN (B-H adjusted *p*-value < 0.05), with at least one compound having |*z*|> 2.0. For each nuclear receptor, the activation/inhibition state (*z*-score) by a compound is indicated and color-coded using a scheme as shown at the bottom right corner

Using more stringent criteria of B-H adjusted *p*-value < 0.05 and |*z*|> 2.0, a total of 5 (1 activated/4 inhibited), 5 (2/3), 6 (5/1) and 3 (0/3) upstream nuclear receptors were identified for CBC, CBD, CBG, and CBN respectively, which are compiled in Fig. 5 with *z*-scores across all the four cannabinoids shown. CBD and CBN were remarkably similar to each other in the activation/inhibition pattern for most of the nuclear receptors. CBC showed similarities with CBD or CBN for many of the inhibited nuclear receptors, while CBG had similar patterns to CBD, and to a lesser extent to CBN, for some of the activated nuclear receptors. Minimal similarities were observed between CBC and CBG. It was also noted that CBG exhibited a much stronger activation pattern (*z* > 2) for several nuclear receptors compared to the other three cannabinoids.

Four nuclear receptors, RARA, ESR1, NR4A1, and NR5A2, were inhibited by all the four cannabinoids. Another three, RXRG, NR4A3, and PPARD, were inhibited only by CBC, CBD or CBN. None of the nuclear receptors were activated by all the cannabinoids. NR0B1 and PGR were activated by CBC, CBD, and CBN, whereas PPARA, NR1D1, NR1I2 and NR1H4 were activated by CBD, CBG and CBN. PPARG and THRB were inhibited by CBC and CBN while activated by CBD and CBG.

### Diseases and biological functions

IPA predicts molecular and cellular functions, physiological system development and function, and diseases and disorders caused by chemical exposure using the resultant DEGs. Over thousands of annotated terms in these categories were enriched (*p* < 0.05) for each of the cannabinoids. The top 1000 for each compound are listed in Supplementary Table 3, and their activation states are shown in Fig. 2C. Unlike CBC, CBD, and CBN, which had more activated (*z* > 0) than inhibited (*z* < 0) entries, the majority of the entries for CBG (with known *z*-scores) were inhibited.

**Fig. 6:**
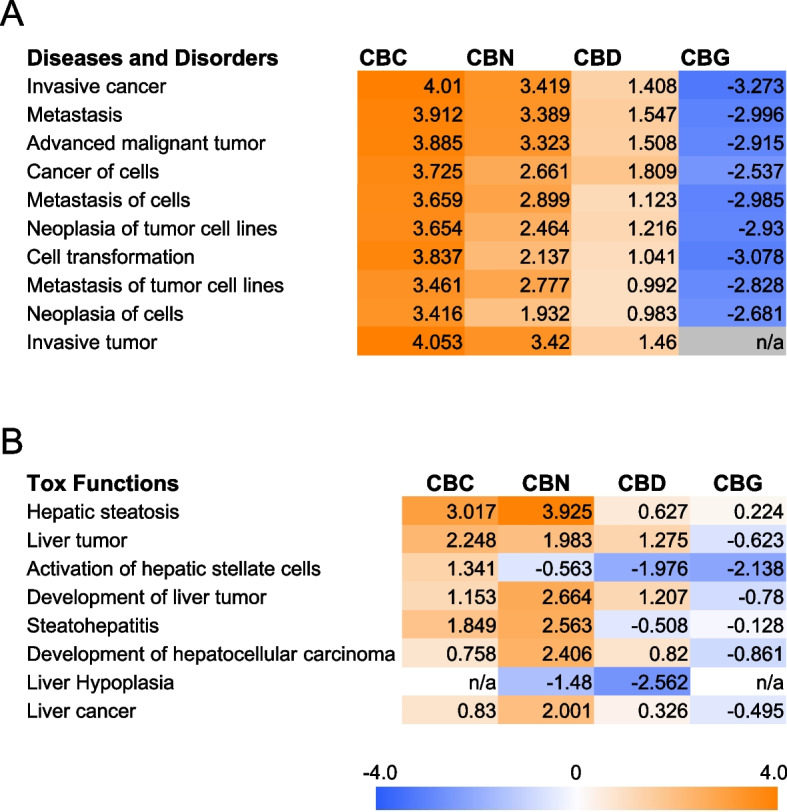
Top 10 diseases and disorders (**A**) and 8 liver tox functions (**B**) identified for CBC, CBD, CBG, and CBN (B-H adjusted *p*-value < 0.05), with at least one compound having |*z*|> 2.0. For each term, the activation/inhibition state (*z*-score) by a compound is indicated and color-coded using a scheme as shown at the bottom right corner

Often, changes in molecular and cellular functions induced by a chemical exposure are the root causes of abnormalities in physiological system development and function, which in turn lead to diseases and disorders as the ultimate result of the exposure. Consequently, heavy redundancies were observed in the enriched terms between the different categories. To streamline the comparison, we focused only on terms within the diseases and disorders category. Using B-H adjusted *p*-value < 0.05, a total of 104 diseases and disorders terms were identified with at least one of the four cannabinoids (Supplementary Table 4) having |*z*|> 2.0. The top 10 are shown in Fig. 6A, which were all related to cancer/tumor development and progression. It was surprising to find that while CBC and CBN strongly activated (*z*-score > 2.0) these terms, CBG strongly inhibited (*z*-score < −2.0) them, except for one term with unknown activation state. CBD also activated these terms but to a less extent (*z*-score < 2.0) than CBC and CBN.

### Toxicological functions

Tox functions in IPA encompass terms in cardiotoxicity, hepatotoxicity, and nephrotoxicity, as well as those in clinical chemistry and hematology assays. A total of 352, 318, 293, and 290 entries were enriched (*p* < 0.05) for CBC, CBD, CBD and CBN respectively. The complete lists of the enriched toxicological function terms with their activation/inhibition states for each cannabinoid are included in Supplementary Table 5 and summarized in Fig. 2D. For all the four cannabinoids, the activation/inhibition state could not be predicted for a large percentage of the enriched tox functions.

We next confined the analysis to hepatotoxicity only. Using B-H adjusted *p*-value < 0.05, only 8 liver tox terms were enriched with at least one of the four cannabinoids having |*z*|> 2.0, (Fig. 6B). Consistent with the findings described in the last section, four functions related to cancer, *liver tumor*, *development of liver tumor*, *development of hepatocellular carcinoma*, and *liver cancer* were activated by CBC, CBD and CBN but inhibited by CBG. All the four cannabinoids activated *hepatic steatosis*, although the activation by CBD or CBG was much weaker compared to CBC and CBN. Interestingly, *steatohepatitis* was also activated by CBC and CBN but weakly inhibited by CBD and CBG. The *activation of hepatic stellate cells* function was activated by CBC only and inhibited by the other three. *Liver hypoplasia* was inhibited by CBD and CBN, but its activation state is unknown for CBC or CBG.

### Biological themes networks

**Fig. 7:**
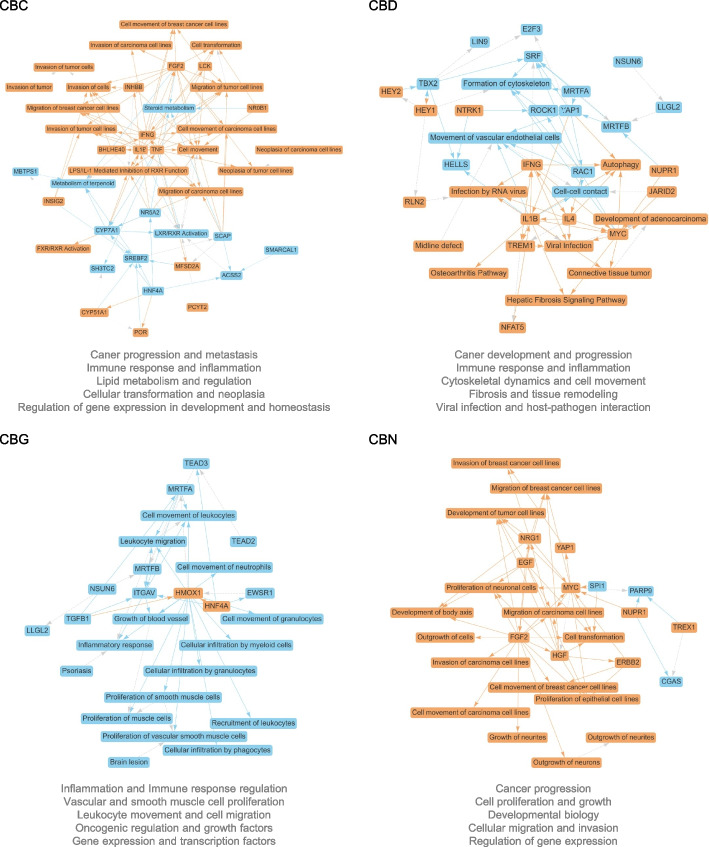
Biological themes networks (graphical summary) of the four cannabinoids. The top 5 biological themes depicted for each compound are listed under the network, of which detailed descriptions are included in Supplementary File 6. The entities (genes, pathways, or functions) in orange color are predicted activation, and those in blue inhibition. Interactions in solid line are direct while those in dotted lines are indirect

To provide a quick overview of the major biological themes in the analysis and to illustrate how those concepts relate to one another, IPA selects and connects a subset of the most significant entities predicted in the analysis (such as canonical pathways, upstream regulators, diseases, and biological functions) to form a network, creating a coherent and comprehensible synopsis of the analysis (*a.k.a.*, graphical summary). The networks for each of the four cannabinoids are shown in Fig. 7, with top biological themes depicted by each network listed under the graph. The central themes for the four cannabinoids are: *cancer progression and metabolic regulation network* for CBC, *interconnected pathways in tumorigenesis and immune response* for CBD, *regulation of inflammation and vascular dynamics through HMOX1 and HNF4A* for CBG, and *EGF-driven cellular dynamics and cancer progression* for CBN. Detailed description of the biological themes can be found in Supplementary Table 6. At first glance, the networks for CBC and CBD showed a roughly balanced mixture of activated and inhibited entities. In contrast, almost all the entities in the CBG network were inhibited whereas the majority of those in the CBN network were activated.

## Discussion

IPA analyses of the cannabinoids transcriptomic data generated sheer amount of information that could not be feasibly described in greater detail. Therefore, we focused the analyses and descriptions on the most prominent canonical signaling pathways, nuclear receptors, diseases and disorders, and tox functions, selected using stringent criteria. Moreover, in addition to identifying common properties among the four different cannabinoids, we also aimed to find distinct features of each compound. In general, CBC, CBD and CBN displayed certain degrees of similarity in the numbers of enriched terms and their activation/inhibition patterns; however, CBG distinguished itself from the other three cannabinoids by its usually smaller numbers and overall inhibition patterns of the enriched canonical pathways, diseases and tox functions (Figs. 2–6).

It needs to be pointed out that IPA uses a default criterion of |*z*|≥ 2 to predict the activation/inhibition state of a pathway, an upstream regulator, or a disease or function. In other words, activated predictions are made only if *z* ≥ 2, and inhibited predictions are made only if *z* ≤ ‑2. IPA does not assign predictions to any values between 2 and ‑2; for example, a prediction is not made for *z* = 1.95. This rather arbitrary default value may render a comparison biased. To make more inclusive and comprehensive comparisons among the cannabinoids, in the current study we make a prediction on the activation/inhibition state as long as a *z*-score is available and non-zero, *i.e.*, activated predictions are made for *z* > 0 and inhibited predictions are made for *z* < 0. However, it needs to be borne in mind that a lower |z| value indicates a less statistically significant (*i.e.*, less reliable) prediction.

The *semaphorin neuronal repulsive signaling pathway* is one of the four canonical signaling pathways commonly activated by all the four cannabinoids (Fig. 3). Semaphorins, originally discovered as guidance cues for developing axons, are involved in many processes that shape the nervous system during development, from neuronal proliferation and migration to neuritogenesis and synapse formation (Carulli et al. 2021). Activation of the pathway may impact neuronal morphology, motility, and connectivity through changes in the cytoskeleton and adhesion, and can contribute to pathological processes in various neurological disorders (Carulli et al. 2021; Jackson and Eickholt 2009; Pasterkamp and Giger 2009). The activation of this pathway by all the four cannabinoids suggests a prevailing adverse effects of long-term consumption of cannabis products may have on nervous system development and plasticity, especially on young adults.

Exposure to the cannabinoids also led to *FXR/RXR activation* (Fig. 3), which refers to the process where the farnesoid X receptor (FXR) and retinoid X receptor (RXR) form a heterodimer and bind to specific DNA sequences to influence gene expression. The pathway activation is crucial for regulating various metabolic processes, including bile acid homeostasis, lipid metabolism, and glucose metabolism (Anderson and Gayer 2021; Tschuck et al. 2023). Interestingly, a related pathway, *LXR/RXR activation*, was inhibited by three of the cannabinoids (CBC, CBD and CBN) and unaffected by CBG (*z* = 0). This pathway activation leads to the formation of the liver X receptor (LXR) and RXR heterodimers which primarily impacts lipid metabolism, inflammation, and cholesterol homeostasis (Bilotta et al. 2020; Fiévet and Staels 2009). The opposite actions of FXR and LXR, as found in the current study, have also been reported in previous studies (Calkin and Tontonoz 2012; Ding et al. 2014). The two receptors work in a complementary and often reciprocal manner to regulate lipid metabolism and bile acid homeostasis, with FXR primarily influencing bile acid synthesis and LXR focusing on lipid transport and cholesterol efflux. Both receptors are implicated in various metabolic disorders and are potential targets for therapeutic interventions (Calkin and Tontonoz 2012; Ding et al. 2014; Kalaany and Mangelsdorf 2006). The final outcome of FXR/RXR activation and LXR/RXR inhibition is unclear; nevertheless, these findings are in line with previous reports that cannabinoids affect metabolism, especially lipid metabolism (Clark et al. 2018; Wiciński et al. 2023). In the case of CBD, preclinical evidence indicates that it modulates lipid metabolism and hepatic steatosis through multiple, context-dependent mechanisms. In murine models of alcohol-induced liver injury, CBD reduced hepatic triglyceride accumulation, inflammation, and oxidative stress (Wang et al. 2017). Similarly, in high-fat/high-cholesterol diet-induced steatohepatitis, CBD attenuated hepatic inflammation and suppressed NF-κB and NLRP3 inflammasome signaling (Huang et al. 2019). At the cellular level, multiomic analyses demonstrate that CBD perturbs cholesterol biosynthesis, transport, and membrane homeostasis pathways (Guard et al. 2022), and experimental macrophage models show reduced foam cell formation with modulation of cholesterol handling (He et al. 2024). However, recent data indicate that CBD can activate pregnane X receptor (PXR), increasing intestinal cholesterol transport and circulating cholesterol in mice, raising potential concerns regarding dyslipidemia under certain conditions (Brown et al. 2024). Clinical data remain limited; in a randomized controlled trial in type 2 diabetes, CBD did not significantly improve lipid profiles relative to placebo (Jadoon et al. 2016). Overall, while existing preclinical findings support biologic plausibility for CBD lipid-modulatory effects, more clinical evidence are needed to confirm its benefit in steatosis or dyslipidemia.

A total of 14 signaling pathways were inhibited by all the four cannabinoids (Fig. 3), among which only one having |z|> or ≈ 2 for each of the cannabinoids – the *atherosclerosis signaling* pathway. Inhibition of the pathway may reduce inflammation and plaque buildup, potentially preventing heart attack and stroke (Kong et al. 2022). However, current findings regarding the effects of cannabinoids on atherosclerosis are inconsistent, and more research is necessitated to clarify the complex relationship between cannabis use and atherosclerosis, including the long-term effects and the specific roles of different cannabinoids (de La Harpe et al. 2023; Guo et al. 2024; Skipina et al. 2022).

Beyond their well-known interaction with CB1 and CB2 receptors, cannabinoids are also known to interact with nuclear receptors. So far, the most studied nuclear receptors in relation to cannabinoids are the peroxisome proliferator-activated receptors (PPARs) (O'Sullivan 2007; Pistis and O'Sullivan 2017). These nuclear receptors play a crucial role in regulating metabolism, inflammation, and gene expression (Kim et al. 2023). Cannabinoids, including phytocannabinoids and endocannabinoids, can activate PPARs, potentially contributing to some of the therapeutic effects attributed to cannabinoids (O'Sullivan 2007; Pistis and O'Sullivan 2017). It was interesting to note that all the three main subtypes of the PPAR family, PPARA, PPARD, and PPARG, were impacted by the cannabinoids; however, their activation/inhibition pattern by the cannabinoids differs with one another. These results suggest that each cannabinoid interacts with the different receptors in a distinctive way, which is consistent with previous findings (O'Sullivan 2007; Pistis and O'Sullivan 2017). Apart from PPARs, evidence suggests that cannabinoids may also interact with other nuclear receptors, though research in this area is limited (Morales and Jagerovic 2020; Pistis and O'Sullivan 2017). The current study provides a dozen or so additional candidates (Fig. 5) for further studying the role of nuclear receptors in cannabinoid function.

Cannabinoids are being investigated for their potential role in both managing cancer symptoms and as a potential cancer treatment. Two synthetic cannabinoids, dronabinol and nabilone, have been approved by the FDA to treat chemotherapy-related nausea and vomiting. Their role in directly combating cancer is still under investigation. Preclinical studies using in vitro and in vivo cancer models have indicated that cannabinoids may have anticancer properties, such as inducing cancer cell death, inhibiting tumor growth, and preventing cancer invasion and metastasis. Several review articles (Dariš et al. 2019; Hinz and Ramer 2022; Tomko et al. 2020; Velasco et al. 2016) covered this topic. It was intriguing to note that the top 10 enriched terms in the diseases and disorders category (Fig. 6A) were all related to cancer/tumor development and progression. Consistent with recently published in vitro studies (Bęben et al. 2024; Kadriya et al. 2024; Lah et al. 2021; Park et al. 2024; Zeppa et al. 2024) showing CBG possesses anticancer activity for a variety of cancer types, CBG was predicted in this study to strongly (|*z*|> 2.5) inhibit 9 of the 10 top cancer-related disease terms (with the remaining one unpredictable). On the other hand, it was surprising to see that, contrary to previous findings on anticancer activity of CBC (Anis et al. 2025), CBD (Bęben et al. 2024; Heider et al. 2022; Mashabela and Kappo 2024; Seltzer et al. 2020), and CBN (Kadriya et al. 2024; Zhong et al. 2023), these three cannabinoids were all predicted to activate all of the top 10 cancer-related disease terms, although the activation by CBD was mild (0.9 < *z* < 1.9) compared to CBC (3.4 < *z* < 4.1) or CBN (1.9 < *z* < 3.5). The reason for these discrepancies is unknown. However, it has to point out that the potential antitumor effects of cannabinoids are predominantly supported by preclinical studies, whereas clinical evidence in humans remains scarce (Skórzewska and Gęca 2024). More importantly, several recent reports indicated genotoxic and pro-cancer effects of cannabinoids or cannabis extract (Fabian-Morales et al. 2021; Kolar et al. 2024; Reece and Hulse 2021; 2023; Reece and Hulse 2024), albeit controversial exists with some other studies showing no significant genotoxic effects (Costa et al. 2025a; Tallon et al. 2025). Therefore, more studies, both preclinical and clinical, are needed to confirm the anti- or pro-cancer effects of various cannabinoids and to elucidate their mechanism(s) of action.

CBD and CBD-rich hemp extracts containing a mixture of cannabinoids and non-cannabinoid phenols, flavonoids, terpenes, alkaloids, and others (ElSohly et al. 2017; Radwan et al. 2021) have been associated with elevated liver enzymes and potential hepatotoxicity in clinical trials (Lo et al. 2023) and more recently in hepatoxicity studies using animal models (Clewell et al. 2023; Costa et al. 2025b; Dehner et al. 2025; Dziwenka et al. 2020, 2023a, 2023b; Ewing et al. 2024; Henderson et al. 2023; Kutanzi et al. 2020; Pinto et al. 2024; Pintori et al. 2024; Polanska et al. 2023) or in vitro models (Campasino et al. 2024; Chen et al. 2024; Gao et al. 2024; Li et al. 2023; Striz et al. 2024). Overall, the results of the in vivo studies indicate that exposure to CBD or CBD-rich hemp extract leads to increases in liver weight and elevation of hepatic-source enzymes in serum (Clewell et al. 2023; Costa et al. 2025b; Dziwenka et al. 2020, 2023a, 2023b; Henderson et al. 2023; Kutanzi et al. 2020), most likely caused by liver cell hypertrophy (Dziwenka et al. 2020, 2023b; Henderson et al. 2023) due to upregulation of hepatic drug metabolizing enzymes (Dziwenka et al. 2020; Ewing et al. 2024; Kutanzi et al. 2020). Recent in vitro studies using various hepatic cell models such as HepG2 (Chen et al. 2024), primary human hepatocytes (Chen et al. 2024; Striz et al. 2024) and induced pluripotent stem cell-derived hepatocytes (Campasino et al. 2024; Gao et al. 2024) demonstrated that CBD causes oxidative stress, cell cycle disturbance, cellular apoptosis, mitochondrial damage, and endoplasmic reticulum (ER) stress in the cells, which might be considered the root causes and early stage events of liver damages. Consistent with these in vivo and in vitro findings, the current transcriptomic study identified several liver tox functions mildly activated (0.3 < *z* < 1.3) by CBD, including *hepatic steatosis*, *liver tumor*, *development of liver tumor*, *development of hepatocellular carcinoma*, and *liver cancer* (Fig. 6B). Two canonical signaling pathways, *NRF2-mediated oxidative stress response* and *unfolded protein response* were both activated by CBD exposure (Fig. 4), suggesting CBD causes cellular oxidative stress in the cells, which may further lead to apoptosis or carcinogenesis.

To date, only a few studies have addressed hepatotoxicity caused by cannabinoids other than CBD (Bailey et al. 2022; Dalterio et al. 1986; Polanska et al. 2023). No hepatotoxicity of CBN was found in a 14-day mouse study (Bailey et al. 2022). However, an early study showed that perinatal exposure to THC, CBN or CBD affects the concentrations of hepatic CYPs in adult male offspring (Dalterio et al. 1986). In addition, CBG causes increases in cellular oxidative stress in rat liver and other hepatotoxic manifestations after a 90-day exposure (Polanska et al. 2023). IPA results on tox functions (Fig. 6B) suggest that CBC and CBN may cause even more severe liver tox than CBD. On the contrary, CBG exhibited slight or mild protective effects to liver toxicity. This is in line with the findings by Polanska et al. (2023). The authors found that, compared with CBD, CBG had opposite effects on the redox state and hepatotoxicity in rats. In fact, throughout the analysis, CBG was found distinct from the other cannabinoids in the activation patterns of the canonical pathways, upstream regulators, diseases and biological functions, toxicity functions, and interaction networks (Figs. 2–7). The exact reason is unclear, but is most likely due to the structural differences between the cannabinoids. CBG is often called the “mother of all cannabinoids” because it is a precursor to the other cannabinoids. The structure of CBG allows it to bind directly to both CB1 and CB2 receptors in the endocannabinoid system. While the other cannabinoids only bind to one of the receptors, or partially or indirectly interact with the receptors (Blebea et al. 2024). Therefore, more studies are needed to fully understand the mechanisms of action of the different cannabinoids, especially CBG.

Several limitations of the present study should be acknowledged. First, the transcriptomic findings were generated using human iPSC-derived hepatocytes, which, although increasingly applied in toxicological investigations (Gao et al. 2023), do not fully recapitulate the architectural complexity, multicellular interactions, and zonation present in the human liver in vivo. iPSC-derived hepatocytes may exhibit differences in maturation state, metabolic enzyme expression, and xenobiotic responsiveness compared with primary human hepatocytes, potentially influencing both the magnitude and spectrum of gene expression changes (Gao and Liu 2017; Gao et al. 2023; Suleman et al. 2025). Second, exposures were conducted for 24 h at concentrations selected to approximate IC_10_ values (Gao et al. 2024) and predicted physiologically attainable liver C_max_ values based on modeling (Liu and Sprando 2023). However, nominal in vitro concentrations may not accurately reflect intracellular or free (unbound) concentrations, nor account for dynamic pharmacokinetic processes such as first-pass metabolism, tissue distribution, or repeated dosing that occur in vivo. Third, pathway enrichment and upstream regulator predictions generated by IPA rely on existing knowledge bases and predictive algorithms, which may introduce bias and do not establish causality. Functional validation at the protein and phenotypic levels is necessary to confirm these transcriptomic predictions. Finally, the study evaluated purified cannabinoids individually, whereas many commercially available products contain complex mixtures of cannabinoids and other phytochemicals (ElSohly et al. 2017; Miller et al. 2022), and potential mixture interactions were not assessed. Therefore, extrapolation of these findings to chronic human exposure and clinical outcomes should be made cautiously, and further in vivo and mechanistic studies are warranted.

## Conclusion

In the current study, we provide a comprehensive transcriptomic functional analysis and comparison of four major cannabinoids found in hemp extract – CBC, CBD, CBG and CBN. Each compound was found to impact a unique list of canonical pathways, upstream regulators, diseases and disorders, toxicity functions, and networks with distinctive activation/inhibition patterns. All the four cannabinoids were predicted to affect metabolism and to have some beneficial effects on cardiovascular disease but adverse effects on the neural system. Similar to but more potently than CBD, CBC and CBN displayed liver toxicity and the potential to cause cancer while CBG protected from these adverse effects. However, further studies are necessitated to confirm these results and to fully understand the mechanisms of action of the different cannabinoids.

## Supplementary Information

Supplementary Material 1.

Supplementary Material 2.

Supplementary Material 3.

Supplementary Material 4.

Supplementary Material 5.

Supplementary Material 6.

## Data Availability

The datasets used and/or analyzed during the current study are available from the corresponding author on reasonable request.
